# Analysis of the kinematic variables that predict jump serve efficacy among volleyball players

**DOI:** 10.1097/MD.0000000000034471

**Published:** 2023-08-04

**Authors:** Mohd Arshad Bari, Ali Azeez Aneed Al Mijbilee, Shibili Nuhmani, Amir Iqbal, Ahmad H. Alghadir

**Affiliations:** a Department of Physical Education, Aligarh Muslim University, Aligarh, Uttar Pradesh, India; b Department of Physical Therapy, College of Applied Medical Sciences, Imam Abdulrahman Bin Faisal University, Dammam, Saudi Arabia; c Department of Rehabilitation Sciences, College of Applied Medical Sciences, King Saud University, Riyadh, Saudi Arabia.

**Keywords:** jump serve analysis, kinematics analysis, multilinear regression, volleyball

## Abstract

In volleyball, a strong correlation exists between the proper application of kinematics factors and the serve results. Therefore, this study compared the kinematics parameters of the volleyball jump serve among different functional classes and established an appropriate multilinear regression model of performance. This correlational observational study involved thirty male collegiate volleyball players categorized into under twenty-three (U-23) men, under twenty-one (U-21) junior men, and under nineteen (U-19) youth boys. Data acquisition entailed the utilization of synchronized cameras to capture the volleyball serves meticulously, while subsequent data analysis was conducted through the implementation of silicon coach–pro 8 motion analysis software. Analysis of variance and multiple linear regressions were performed to analyze data, with a predetermined significance level of *P* < .05. Jump serve analysis showed significant mean differences in selected major kinematic variables among all 3 classes (U-23 men, U-21 junior men, and U-19 youth boys). U-23 men Model-3, which includes 3 independent variables (approach velocity [AV], shoulder extension angles during the cocking phase, and center of gravity [CG] height), predicted velocity with an R-square of 1.00, indicating that the selected independent variable caused 100% variation in ball velocity (BV), whereas models 1 and 2 showed 99% variation in BV, respectively. The U-21 Junior men Model-2, which includes 2 independent variables (height of CG and shoulder extension angles during the cocking phase), predicted velocity with an R-square of 9.80, indicating that the selected independent variable caused a 98% variation in BV. In contrast, model 1 showed a 94% variation in BV, respectively. U-19 youth boys Model-1, which includes one independent variable (AV), predicted velocity with an R-square of 0.89, indicating that the selected independent variable caused 89% variation in BV. The jump serve exhibits similar biomechanical characteristics across different classes. However, the major independent variables of the jump serve: U-23 men were AV, shoulder extension angles at cocking phase (SEACP), the height of CG, U-21 junior men were SEACP and height of CG, and U-19 youth boys were SEACP and height of CG AV showed significant with the dependent variable (BV).

## 1. Introduction

Volleyball, a widely recognized sport, was first introduced by William G. Morgan in 1895 and has since gained significant popularity worldwide. Throughout its history, the sport has evolved in terms of rules and formats,^[1]^ further establishing its significance, particularly with its inclusion in the 1964 Olympic Games. Volleyball is a team-oriented sport that emphasizes both offensive and defensive skills, with the primary goal being the strategic placement of the ball in the opposing team court. Among the various actions in the game, the volleyball service holds particular importance in gaining a competitive advantage over opponents. Achieving successful serves requires precise tactics and pacing, with several kinematic factors, including upper body kinematics, approach distance, approach velocity (AV), and the height of the center of gravity (CG), influencing the flight distance and velocity of the ball.^[2,3]^ Applying these kinematic factors in the serve demonstrates strong correlations with serve outcomes.^[4–6]^

Continuous participation in sports activities can decrease player intensity and passion over time.^[7]^ To maintain optimal performance levels, it is crucial to strike a balance between the game demands and the players physical fitness. In recent decades, scientific training programs have emerged as essential components for the advancement and success of volleyball. Regarding the serve, variations can be observed, such as standing or jump serves. Jump serves, particularly those executed with topspin, involve the player jumping in court and striking the ball towards the opponent court from the baseline.^[8]^ Due to the aerodynamics influenced by topspin and side spin, the serve imparts unique movements to the ball, making it challenging for the receiver to handle effectively and reducing the chances of an easy reception. Jump and spin serves exhibit distinct vertical and horizontal ball movements with varying speeds. Controlling the spin on the ball plays a crucial role in the game of volleyball. The server objective is to deliver the ball within the court while considering factors such as directional control, speed, and acceleration to make it difficult for the receiver to handle it effectively. To achieve this, the server must consider elements like the angle of the arm and the level of hand-ball striking, which influence the serving distance and maintain a competitive level of proficiency.^[8–10]^

The volleyball serve represents a dynamic art form that varies based on individual requirements, skills, and specific match situations.^[11]^ The effectiveness of the serve often proves to be a determining factor in the outcome of a game, as elite players can generate high ball speeds, limiting their opponents attacking capabilities. While different serving methods exhibit similar maximum speed ranges, power serves tend to have higher mean speeds.^[12]^ Previous scientific analyses have emphasized the utilization of the jump serve as a means for volleyball players to achieve successful serves and gain an advantage in scoring points. Serve velocity plays a significant role in securing an upper hand in volleyball.^[13]^ Thus, this study focuses on the speed and kinematic parameters of the volleyball jump serve as the dependent and independent variables, respectively. Numerous biomechanical analysis studies have been conducted on the volleyball serve, primarily in coaching, training, and major tournaments. However, a comprehensive understanding of the biomechanical aspects specifically related to the volleyball jump serve is still lacking.^[14–17]^

Despite its extensive historical background, volleyball has received insufficient attention in terms of biomechanical analysis. The biomechanical examination of volleyball motion focuses on key variables such as velocity, trajectory angle, performance mechanics, and correlation, collectively known as “digitization.” These variables allow for a comprehensive evaluation of serve effectiveness, which can be measured through various methods. Prioritizing speed in volleyball is crucial for minimizing errors and gaining a competitive advantage over opponents. Unfortunately, there is a lack of comparative studies on these biomechanical procedures across different functional classes. This research aims to comprehensively explore various aspects of the jump serve, including its execution, the influence of independently selected factors on ball velocity (BV), the performance of different age groups, and disparities in performance, providing insights into the biomechanics of this particular skill.

## 2. Methods

### 2.1. Study design

The study was based on an observational research design, aiming to analyze the kinematic variables that can predict the efficacy of the jump serve among collegiate volleyball players.

### 2.2. Ethical considerations

The research method was carried out in accordance with the declaration of Helsinki on ethical principles for medical research involving human participants and was approved by the ethics committee, Aligarh Muslim University, Aligarh, India (ethical approval number: PE/MAB/2017/2). Before starting the study, a signed informed-consent form was obtained from each participant and their legal guardians (in the case of minor participants aged below 16 years).

### 2.3. Setting

Collegiate students who were playing volleyball regularly were invited using posters and pamphlets to participate voluntarily in the volleyball ground of the university. This study took 3-month to complete after its beginning in March 2018.

### 2.4. Participants

Thirty (30) male collegiate volleyball players volunteered to take part in this study. According to their age, the groups were classified into 3 separate subcategories (under twenty-three [U-23] men, under twenty-one [U-21] junior men, and under nineteen [U-19] youth boys). To confirm that each athlete was eligible for the research, a brief medical history was obtained from participants. None of the players ever had elbow or shoulder surgery, and none of them had ever complained of pain while serving. A written signed informed consent was obtained from each participant before the beginning of the study.

### 2.5. Selection of variables

Following an assessment of the relevant literature and consultation with subject professionals, the determinants for jump serve were chosen. Variables considered for serve were height, weight, age, shoulder extension angle and elbow extension angle at cocking phase, maximum elbow extension and maximum shoulder extension at acceleration phase, maximum elbow extension angular velocity, maximum shoulder internal rotation/flexion angular velocity, wrist, shoulder and elbow angles and wrist angular velocity at ball contact phase, spike height, takeoff to line distance, approach distance, AV, height of CG and BV.

### 2.6. Data collection and analysis

**Figure 1.**
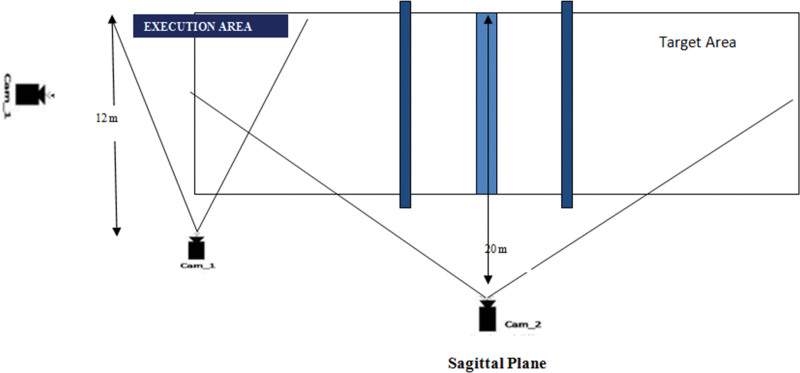
Experimental. setup.

The subject volleyball serves were recorded using 3 synchronized Nikon D-7000 video cameras in a field setting. The monitor was set to sports mode and the video camera sampling rate was set to one hundred twenty (120) fields per second and 1/2000 fast shutter speed. The 1-camera was located 12 (m) perpendicular to the sagittal line and parallel to the mediolateral axis as their volleyball serve hand, resulting in a 90^o^ angle between their respective optical axes. The second camera was positioned perpendicular to the sagittal plane on the right side of the volleyball pole, 20 meters apart, to measure BV and trajectory. The third camera is placed just behind the volley ball server. Additionally, the cameras were raised (1.50 m) and rotated in order to obtain the largest picture possible while keeping all points of interest completely within movement Figure 1. Three trials were completed by the subjects, and one of the finest trials was chosen for further analysis. Along the service line, a calibration band (1.21 m by 1.21) was recorded. Twenty-three passive markers were digitized and evaluated with the silicon coach pro 8 motion analysis program, according to Davis protocols. The digitizing procedure began with 06 (six) video frames just before initiating the movement and finished with 05 frames after ball impact.

### 2.7. Statistical analysis

A statistical tool IBM SPSS Statistics for Windows, version 23 (IBM Corp., Armonk, NY) was used for the statistical analysis.^[18]^ Analysis of variance and multi linear regression analysis were used to investigate differences in kinematic characteristics of distinct functional classes, as well as the established predictive model of serve performance. The significance level was set at *P* < .05 for all statistical analysis in this study.

## 3. Results

The descriptive characteristics of the participants are available as Table 1.

**Table 1:** The descriptive characteristics of the participants.

Characteristics	U-23 Men	U-21 Junior men	U-19 Youth boys
Age (yr)	22.20 ± 0.41	19.40 ± 3.51	17.40 ± 0.69
Height (cm)	182.60 ± 4.00	180.70 ± 2.35	179.00 ± 1.88
Weight (kg)	80.70 ± 7.46	80.10 ± 3.38	66.90 ± 1.88

U-19 = under nineteen, U-23 = under twenty-three.

Table 2 list the selected kinematics variables of jump serve during serve execution. The U-23 men have greater values on shoulder extension angle and elbow extension angle at cocking phase, maximum elbow extension and maximum shoulder extension at acceleration phase, maximum shoulder internal rotation/flexion angular velocity, shoulder and elbow angles and wrist angular velocity at ball contact phase, spike height, takeoff to line distance, approach distance, AV, height of CG and BV as compared with other age groups.

**Table 2:** Descriptive statistics of kinematics variable of the jump serve.

Variable		Mean (standard deviation)
Shoulder extension angles at cocking phase (SEACP) ^O^	U-23 Men	2.70 (0.75)
	U-21 Junior men	2.28 (0.53)
	U-19 Youth boys	2.21 (0.46)
Elbow extension angles at Cocking Phase (EEACP) ^O^	U-23 Men	1.39 (0.38)
	U-21 Junior men	1.31 (0.21)
	U-19 Youth boys	1.31 (0.18)
Maximum elbow extension angular velocity (MEEAV) ^0^/s	U-23 Men	1561.20 (123.68)
	U-21 Junior men	1622.80 (141.93)
	U-19 Youth boys	1589.30 (128.27)
Maximum shoulder flexion angular velocity (MSFAV)^0^/s	U-23 Men	2484.40 (208.24)
	U-21 Junior men	2364.90 (231.93)
	U-19 Youth boys	2119.70 (96.38)
Wrist angle at ball contact (SABC)^0^	U-23 Men	164.20 (20.61)
	U-21 Junior men	172.50 (6.39)
	U-19 Youth boys	152.20 (15.41)
Elbow angle at ball contact (EABC)^0^	U-23 Men	133.10 (3.41)
	U-21 Junior	128.20 (6.23)
	U-19 Youth boys	119.80 (2.04)
Shoulder angle at ball contact (SABC)^0^	U-23 Men	71.10 (8.83)
	U-21 Junior men	65.20 (9.86)
	U-19 Youth boys	59.20 (10.13)
Wrist angular velocity at ball contact (WAVBC)^0/s^	U-23 Men	251.90 (15.44)
	U-21 Junior men	242.90 (19.39)
	U-19 Youth boys	245.90 (20.42)
Spike height (cm)	U-23 Men	327.40 (3.77)
	U-21 Junior men	268.80 (24.67)
	U-19 Youth boys	287.60 (30.19)
Take-off to line distance (TLD) cm	U-23 Men	83.40 (10.09)
	U-21 Junior men	82.80 (9.25)
	U-19 Youth boys	76.80 (6.95)
Approach distance (m)	U-23 Men	3.17 (0.32)
	U-21 Junior men	3.09 (0.26)
	U-19 Youth boys	2.70 (0.17)
Approach Velocity (AV) m/s.	U-23 Men	2.82 (0.33)
	U-21 Junior men	2.61 (0.17)
	U-19 Youth boys	2.60 (0.07)
Height of CG	U-23 Men	1.72 (0.06)
	U-21 Junior men	1.61 (0.13)
	U-19 Youth boys	1.63 (0.07)
Ball velocity (m/s)	U-23 Men	22.70 (3.27)
	U-21 Junior men	21.57 (2.06)
	U-19 Youth boys	19.99 (1.98)

AV = approach velocity, CG = center of gravity, U-19 = under nineteen, U-21 = under twenty-one, U-23 = under twenty-three.

Table 3 list the significant mean differences among U-23 men, U-21 junior men and U-19 youth boys volleyball players on maximum shoulder flexion angular velocity o/s at acceleration phase, wrist angle at ball contact phase, elbow angle at ball contact phase, shoulder angle at ball contact phase, spike height, approach distance and height of CG (*P* < .05). At the same time, shoulder extension angles at the cocking phase, elbow extension angles at cocking phase, maximum elbow extension angular velocity (^o^/s), wrist angular velocity(^o^/s) at ball contact phase, take off to line distance (cm), AV (m/s) and BV (m/s) showed insignificant mean differences.

**Table 3:** One-way analysis of variance (ANOVA) in upper body kinematics across the groups.

ANOVA		Sum of squares	df	Mean square	Partial eta squared	F-value	Sig.
Shoulder extension angles at Cocking Phase (SEACP)	Between groups	1.36	2	0.68	0.12	1.91	0.16
	Within groups	9.59	27	0.35			
	Total	10.95	29				
Elbow extension angles at cocking phase (EEACP)	Between groups	0.038	2	0.02	0.01	0.25	0.78
	Within groups	2.019	27	0.07			
	Total	2.057	29				
Maximum elbow extension angular velocity (MEEAV) ^0^/s	Between groups	19021.40	2	9510.70	0.03	0.55	0.58
	Within groups	467037.30	27	17297.67			
	Total	486058.70	29				
Maximum shoulder flexion angular velocity (MSIRAV)^0^/s	Between groups	691364.60	2	345682.30	0.41	9.74*	0.00
	Within groups	958013.40	27	35481.97			
	Total	1649378.00	29				
Wrist angle at ball contact phase (WABC)^0^	Between groups	2083.26	2	1041.63	0.24	4.44*	0.02
	Within groups	6333.70	27	234.58			
	Total	8416.97	29				
Elbow angle at ball contact phase (EABC)^0^	Between groups	904.86	2	452.43	0.64	24.82*	0.00
	Within groups	492.10	27	18.22			
	Total	1396.96	29				
Shoulder angle at ball contact phase (SABC)^0^	Between groups	708.06	2	354.03	0.22	3.82*	0.03
	Within groups	2502.10	27	92.67			
	Total	3210.16	29				
wrist angular velocity at ball contact phase (WAVBC) ^0/s^	Between groups	420.00	2	210.00	0.04	0.61	0.55
	Within groups	9284.70	27	343.87			
	Total	9704.70	29				
Spike height (cm)	Between groups	17904.80	2	8952.40	0.56	17.50*	0.00
	Within groups	13812.40	27	511.57			
	Total	31717.20	29				
Takeoff to line distance (TLD) cm	Between groups	266.40	2	133.20	0.11	1.69	0.20
	Within groups	2123.60	27	78.65			
	Total	2390.00	29				
Approach distance (m)	Between groups	0.86	2	0.43	0.31	6.37*	0.00
	Within groups	1.83	27	0.07			
	Total	2.70	29				
Approach velocity (m/s)	Between groups	0.30	2	0.15	0.19	3.24	0.06
	Within groups	1.26	27	0.05			
	Total	1.57	29				
Height of CG (m)	Between groups	0.07	2	0.03	0.24	4.02*	0.03
	Within groups	0.22	27	0.01			
	Total	0.29	29				
Ball velocity m/s	Between groups	37.11	2	18.56	0.17	2.96	0.07
	Within groups	169.42	27	6.27			
	Total	206.54	29				

CG = center of gravity.

* Significant value if *P* < .05.

### 3.1. A description of the multiple linear regression analysis of volleyball players jump serve

Multiple linear regression analysis was used to create regression modules for the jump serves of U-23 men, U-21 junior men, and U-19 youth boys volleyball players. Out of 14 selected kinematic variables, volleyball players U-23 men shoulder extension angles at cocking phase (SEACP) and height of CG, U-21 junior men SEACP and height of CG and U-19 youth AV were entered by using a stepwise multiple regression module with a dependent variable of BV (Criteria: Probability-of-F-to-enter ≤ 00.05, Probability-of-F-to-remove ≥ 00.10).

Table 4 lists the Under-23 men Model 1, which incorporates only 1 parameter (AV), model 2 which incorporates only 2 parameters (AV, SEACP) and model 3, which incorporates 3 parameters (AV, SEACP, and height of CG), predicts velocity with R squares of 0.98, 0.98, and 1.00, indicating that the variables account for 98%, 98% and 100% differences in velocity of volleyballUnder-21junior men Model 1, which incorporates only 1 parameter (height of CG) and Model 2, which incorporates 2 parameters (height of CG and SEACP), predict velocity with an R square of 0.94 and 0. 98 (percent) indicating that the variable accounts for 94% and 98% (percent) differences in velocity of volleyball. Under-19 youth boys Model 1, which incorporates only 1 parameter (AV), predicts velocity with an R square of 0.89, indicating that the variable accounts for 89% (percent) difference in velocity of volleyball.

**Table 4:** Summary of regression models of volleyball serves.

	Model	*R*	*R* ^2^	Adjusted *R*^2^	Std. error of the estimate
U-23 men	1	00.99†	00.99	00.98	00.38
	2	01.00‡	00.99	00.95	00.28
	3	00.99§	01.00	01.00	00.21
†Predictors: (Constant), approach velocity ‡Predictors: (Constant), approach velocity (AV), shoulder extension angles at cocking phase §Predictors: (Constant), approach velocity (AV), shoulder extension angles at cocking phase, the height of CG.					
U-21 Junior men	1	00.97†	00.94	00.93	00.55
	2	00.99‡	00.98	00.97	00.36
†Predictors: (Constant), the height of CG ‡Predictors: (Constant), the height of CG, shoulder extension angles at cocking phase.					
U-19 Youth boys	1	00.94†	00.89	00.86	00.70
†Predictors: (Constant), approach velocity.					

U-19 = under nineteen, U-21 = under twenty-one, U-23 = under twenty-three.

Table 5 lists the regression equations based on models 1, 2, and 3 were significant (*P* = .00, *P* < .05), as seen in Table 3. As a result of the study, U-23 men volleyball players BV can be estimated/predicted by using AV in model-1, AV, SEACP in model-2, and AV, SEACP, height of CG in model 3.

**Table 5:** Significance of the regression model (U-23 Men volleyball players jump serve).

ANOVA†						
Model		Sum of Squares	df	Mean square	F	Sig.
1	Regression	94.95	1	94.95	664.51*	00.00‡
	Residual	01.13	8	00.13		
	Total	96.17	9			
2	Regression	95.45	2	47.72	526.58*	00.00§
	Residual	00.63	7	00.08		
	Total	96.91	9			
3	Regression	95.82	3	31.93	713.20*	00.00∥
	Residual	00.26	6	00.04		
	Total	96.09	9			

ANOVA = analysis of variance.

† Dependent Variable: ball velocity.

‡ Predictors: (Constant), approach velocity.

§ Predictors: (Constant), approach velocity, shoulder extension angles at cocking phase.

∥ Predictors: (Constant), approach velocity, shoulder extension angles at cocking phase, the height of CG.

* Significant value if *P* < .05.

The regression equations based on models 1, and 2 were significant (*P* = .00, *P* < .05), as seen in Table 6. As a result of the study, U-21 junior men volleyball players BV can be estimated/predicted by the height of CG in model-1 and the height of CG, and SEACP in model-2.

**Table 6:** Significance of the regression model (U-21 junior men volleyball players jump serve).

ANOVA†						
Model		Sum of squares	df	Mean square	F	Sig.
1	Regression	35.657	1	35.652	120*	0.00‡
	Residual	2.384	8	0.29		
	Total	38.041	9			
2	Regression	37.135	2	18.563	144*	0.00§
	Residual	0.906	7	0.122		
	Total	38.041	9			

ANOVA = analysis of variance.

† Dependent Variable: ball velocity.

‡ Predictors: (Constant), the height of CG.

§ Predictors: (Constant), the height of CG, shoulder extension angles at cocking phase.

* Significant value, if *P* < .05.

The regression equation based on model-1 was significant (F = 64.09, *P* = .00, *P* < .05), as seen in Table 7. As a result, U-19 youth boys volleyball players BV can be estimated/predicted by using the AV.

**Table 7:** Significance of the regression model (U-19 youth boys volleyball players jump serve).

ANOVA†						
Model		Sum of squares	df	Mean square	F-value	*P* value
1	Regression	31.371	1	31.363	64*	.00*^,^‡
	Residual	3.924	8	0.492		
	Total	35.283	9			

ANOVA = analysis of variance, U-19 = under nineteen.

† Dependent variable: ball velocity;

‡ Predictors: (Constant), approach velocity.

* Significant value if *P* < .05.

The Regression equation is estimated on the basis of the above-mentioned Table 8 as following:

**Table 8:** Coefficients of the regression model volleyball players jump serve.

	Coefficients†						
	Model		Unstandardized coefficients		Standardized coefficients	*t* value	Sig.
			Beta	Std. Error	Beta		
U-23 Men	1	(Constant)	−5.19	1.08		−4.76*	0.00
		Approach velocity	9.90	0.37	0.99	25.77*	0.00
	2	(Constant)	−5.91	0.86		−5.86*	0.00
		Approach velocity	10.17	0.32	1.01	30.93*	0.00
		Shoulder extension angles at cocking phase	−0.33	0.13	−0.08	−2.36*	0.03
	3	(Constant)	2.31	2.6		0.86	0.41
		Approach velocity	10.47	0.26	1.05	41.13*	0.00
		Shoulder extension angles at cocking phase	−0.55	0.12	−0.13	−4.39	0.01
		Height of CG	−4.49	1.57	−0.08	−2.85	0.03
U-23 Junior men	1	(Constant)	−3.46	2.29		−1.50	0.17
		Height of CG	15.55	1.42	0.96	10.91	0.00
	2	(Constant)	−2.35	1.54		−1.52	0.17
		Height of CG	15.96	0.94	0.99	16.89*	0.00
		Shoulder extension angles at cocking phase	−0.76	0.22	−0.19	−3.38	0.01
U-19 Youth boys	1	(Constant)	−50.85	8.85		−5.75	0.00
		Approach velocity	27.23	3.40	0.94	8.01*	0.00

U-19 = under nineteen, U-23 = under twenty-three.

* Significant value, if *P* < .05.

† Dependent variable ball velocity.

U-23 men Model-1. BV = (9.9) AV—5.19

Here, the AV coefficient was found statistically significant using with (*P* = .00, *P* < .05).

U-23 men Model-2. BV = (10.17) AV + (−0.33) SEACP − 5.91

Here, the AV and SEACP coefficient were found statistically significant using with (*P* = .00, and *P* = .03, *P* < .05).

1. BV has a positive effect with AV, when SEACP is kept constant.
2. BV has a negative effect with SEACP, when AV is kept constant.

U-23 men Model-3. BV = (10.47) AV + (−0.55) SEACP + (−4.49) height of CG + 2.31

Here, the AV and SEACP and height of CG coefficient were found statistically significant using with (*P* = .00, and *P* = .03, *P* = .03 *P* < .05).

1. BV has a positive effect with AV, when SEACP and height of CG are kept constant.
2. BV has a negative effect with SEACP when, AV and height of CG are kept constant.
3. BV has a negative effect with height of CG, when AV and SEACP are kept constant.

U-21 junior men Model-1. BV = (15.55) height of CG–3.46

Here, the height of CG coefficient was found statistically significant using with (*P* = .00, *P* < .05).

U-21 junior men Model-2. BV = (15.96) height of CG + (–0.76) SEACP − 2.35

Here, the height of CG and SEACP coefficient were found statistically significant using with (*P* = .00, and *P* = .01, *P* < .05).

1. BV has a positive effect on the height of CG, when shoulder extension angle at cocking phase is kept constant.
2. BV has a negative effect on SEACP, when the height of CG is kept constant.

U-19 youth boy Model-1. BV = (27.23) AV − 50.85

Here, the AV coefficient was found statistically significant using with (*P* = .00, *P* < .05).

### 3.2. Selected regression models

U-23 Men model: BV = (10.47) AV + (−0.55) SEACP + (−4.49) height of CG + 2.31

U-21 junior men model: BV = (15.96) height of CG + (−0.76) SEACP − 2.35

U-19 youth boys Model: BV = (27.23) AV − 50.85

## 4. Discussion

Volleyball is a dynamic game that can differ due to individual needs, skills and match situations. This intervention tends to be a decisive factor in the outcome of a certain game. Thus, at a high stage, the player can generate a high ball speed to limit their opponents attack capabilities. The findings of the study revealed that selected performance models were statistically significant. U-23 men Model-3, which includes 3 independent variables (AV, shoulder extension angles during the cocking phase, and the CG height), predicted velocity with an R-square of 1.00, indicating that the selected independent variable caused 100% variation in BV. In contrast, models 1 and 2 showed 99% variation in BV, respectively. U-21 junior men Model-2, which includes 2 independent variables (the height of CG and shoulder extension angles during the cocking phase), predicted velocity with an R-square of 0.98, indicating that the selected independent variable caused 98% variation in BV, whereas models 1 showed 94% variation in BV, respectively. U-19 youth boys Model-1, which includes 1 independent variable (AV), predicted velocity with an R-square of 0.89, indicating that the selected independent variable caused 89% variation in BV.

In the present study, BV has also been improved but not significantly as the efficiency standard has increased. The average score for serving velocity among men U-23 men, U-21 junior men, and U-19 youth volleyball players in categories was 22.70 m/s, 21.57 m/s, and 19.99 m/s. During the execution volleyball serve, a taller athlete has an advantage compared to other athletes with shorter height because taller athletes have higher ball contact and greater height of ball release. In volleyball, serve height of ball contact very important factor, which have a greater angle of release.^[19–21]^ Ball position at impact is determined by height of the CG, spike height and toss of the server- a successful serve requires a perfect place of toss and contact angle of the serve is required a higher BV.

Analysis of jump serve showed significant mean differences in selected major kinematic variables among the 3 classes in line with the previous studies were shown significant mean differences in upper body kinematics between different functional classes and elbow joint has significant relation with BV.^[22]^ Few studies conducted on volleyball jump serve and found that elbow and humerus extensions were significant factors while jump serve action.^[14,20,23]^ According to previous scientific examinations, the ball should be serving around the body midline to have contact with the hitting shoulder.^[24]^ The volleyball is contacted with a relaxed hand just as the elbow completes its extension. The wrist is flexing and the hand is rotating forward due to pronation and wrist adduction.^[25]^ The velocity of the hand at contact is one of the most important variables in the jump serve, as the faster the hand velocity the faster the BV. The ball will always leave the hand at a faster velocity than that of the hand, due to the transfer of momentum from the heavier hand and arm to the lighter volleyball. In current analysis U-23 men maximum shoulder flexion angular velocity reported higher in comparison with other junior groups. In line with the previous examinations, the BV in jumps serve was noticeably higher (23.03 ± m/s) in the senior age group (23.7 m/s).^[14]^ It was clearly indicated that arm traveled speed play significance role in serve velocity. A study of the serve of collegiate male volleyball players revealed that the players hand traveled at 13.6 m/s prior to contact, while the ball left the hand at 19.7 m/s.^[26]^ When compared to the spike in which the player hand traveled at 15.4 m/s and the ball left the hand at 22.4 m/s, suggesting that the velocity of the hand is close to 70% of the velocity of the ball after impact.^[26]^

BV in all serve has same pattern that senior players has more speed as compare with junior player. Senior players have more power, strength and have the skill hitting the ball more accurately in front of hitting shoulder as compare with juniors. Complex skill that is currently performed by almost all highly skilled volleyball players at all levels of play. Adherence to the suggestions included here describing the movements and timing involved in ideal technique will improve the skill of players at all levels. Even if these extreme positions are difficult for players of lower strength and flexibility levels, attempting to increase range of movement, joint movement timing and jump height will improve jump serve effectiveness.

## 5. Limitation

The study is limited by a small sample size, making it difficult to generalize the findings to other populations and skill levels. The observational design prevents establishing causal relationships, and the absence of a control group limits the ability to compare results. Reliance on a single outcome measure, BV, may overlook other important aspects of performance. The study controlled laboratory conditions may not fully capture the complexities of real-game situations, and alternative statistical approaches could provide additional insights. The study does not consider the anthropometrical and temporal aspects of kinematic variables or include a comprehensive set of variables.

## 6. Scope

The study findings suggest that coaches and trainers can utilize factors such as AV, shoulder extension angles, and CG height to design training programs that enhance the jump serve performance of volleyball players. An individualized coaching approach, tailored to each player strengths and weaknesses, is recommended to maximize improvement potential and optimize overall performance. Biomechanical analysis techniques, such as motion capture systems and video analysis, can provide real-time assessment and feedback on kinematic variables, enabling players to make necessary adjustments and optimize their jump serve. Additionally, the identification of key kinematic variables associated with successful jump serves can inform talent identification and player selection processes, leading to the development of standardized assessment protocols. Incorporating these recommendations in future studies and practical applications has the potential to advance volleyball training, performance analysis, and talent development.

## 7. Suggestion and recommendations

Further research is justified to investigate additional variables and factors, such as footwork kinematics, arm swing mechanics, and body positioning, that have the potential to influence the performance of the volleyball jump serve. Conducting similar studies with female volleyball players would provide a more comprehensive understanding of the underlying kinematic factors and facilitate a comparative analysis of biomechanical characteristics and performance predictors across genders. Moreover, longitudinal studies that track the evolution of kinematic parameters and performance outcomes over an extended duration hold promise in offering valuable insights into the developmental trajectory of the jump serve and identifying potential prognostic indicators for performance enhancement.

## 8. Conclusion

In conclusion, jump serve acts similar in terms of biomechanical characteristics of volleyball serve. But the major independent variables of jump serve: U-23 men were AV, SEACP, the height of CG, U-21 junior men were SEACP and the height of CG, and U-19 youth boys were SEACP and the height of CG AV showed significant with dependent variable (BV). The jump serve is an exciting and complex skill that is currently performed by almost all highly skilled volleyball players at all levels of play. Adherence to the suggestions included here describing the movements and timing involved in ideal technique will improve the skills of players at all levels. Even if these extreme positions are difficult for players of lower strength and flexibility levels, attempting to increase range of movement, joint movement timing and jump height will improve jump serve effectiveness.

## Author contributions

**Conceptualization:** Mohd Arshad Bari, Ali Azeez Aneed Al Mujbilee, Shibili Nuhmani.

**Data curation:** Mohd Arshad Bari, Ali Azeez Aneed Al Mujbilee.

**Formal analysis:** Mohd Arshad Bari, Amir Iqbal.

**Funding acquisition:** Ahmad H. Alghadir.

**Investigation:** Mohd Arshad Bari, Ali Azeez Aneed Al Mujbilee.

**Methodology:** Mohd Arshad Bari, Ali Azeez Aneed Al Mujbilee.

**Project administration:** Mohd Arshad Bari, Ali Azeez Aneed Al Mujbilee, Shibili Nuhmani, Amir Iqbal.

**Resources:** Amir Iqbal, Ahmad H. Alghadir.

**Supervision:** Shibili Nuhmani, Ahmad H. Alghadir.

**Validation:** Shibili Nuhmani.

**Writing – original draft:** Mohd Arshad Bari, Ali Azeez Aneed Al Mujbilee, Amir Iqbal.

**Writing – review & editing:** Mohd Arshad Bari, Shibili Nuhmani, Amir Iqbal, Ahmad H. Alghadir.
